# Staphylococcus aureus Lipase 1 Enhances Influenza A Virus Replication

**DOI:** 10.1128/mBio.00975-20

**Published:** 2020-07-07

**Authors:** Mariya I. Goncheva, Carina Conceicao, Stephen W. Tuffs, Hui-Min Lee, Marlynne Quigg-Nicol, Ian Bennet, Fiona Sargison, Amy C. Pickering, Saira Hussain, Andrew C. Gill, Bernadette M. Dutia, Paul Digard, J. Ross Fitzgerald

**Affiliations:** a The Roslin Institute, University of Edinburgh, Midlothian, United Kingdom; Icahn School of Medicine at Mount Sinai

**Keywords:** *Staphylococcus aureus*, influenza, influenza vaccines, lipase, pathogenesis

## Abstract

Influenza A virus (IAV) causes annual epidemics of respiratory disease in humans, often complicated by secondary coinfection with bacterial pathogens such as Staphylococcus aureus. Here, we report that the S. aureus secreted protein lipase 1 enhances IAV replication *in vitro* in primary cells, including human lung fibroblasts. The proviral activity of lipase 1 is dependent on its enzymatic function, acts late in the viral life cycle, and results in increased infectivity through positive modulation of virus budding. Furthermore, the proviral effect of lipase 1 on IAV is exhibited during *in vivo* infection of embryonated hen’s eggs and, importantly, increases the yield of a vaccine strain of IAV by approximately 5-fold. Thus, we have identified the first S. aureus protein to enhance IAV replication, suggesting a potential role in coinfection. Importantly, this activity may be harnessed to address global shortages of influenza vaccines.

## Abstract (precis)

Influenza A virus (IAV) causes annual epidemics and sporadic pandemics of respiratory disease. Secondary bacterial coinfection by organisms such as Staphylococcus aureus is the most common complication of primary IAV infection and is associated with high levels of morbidity and mortality. Here, we report the first identified S. aureus factor (lipase 1) that enhances IAV replication during infection via positive modulation of virus budding. The effect is observed *in vivo* in embryonated hen’s eggs and greatly enhances the yield of a vaccine strain, a finding that could be applied to address global shortages of influenza vaccines.

## INTRODUCTION

Influenza A virus (IAV) is a member of the *Orthomyxoviridae* family, with a segmented, negative-sense RNA genome. Aquatic birds are viewed as the reservoir host, but it infects a wide variety of vertebrate hosts, including birds, bats, and terrestrial and aquatic mammals (1). The virus is antigenically diverse and across all hosts has at least 18 subtypes of the major surface glycoprotein, hemagglutinin (HA), and 11 of the lower-abundance neuraminidases (NA) (2). A common feature of all HA subtypes is that the molecule is synthesized as a precursor (HA_0_) that, after assembly into a trimer, must be proteolytically cleaved into HA_1_ and HA_2_ subunits to produce infectious virus particles (3). This cleavage step is achieved in cell culture by the addition of trypsin to the media, while secreted trypsin-like proteases of respiratory or mucosal epithelia perform this role in human seasonal IAV infections or low-pathogenicity avian influenza virus infections (4). Virus replication is entirely dependent on this step, making it an attractive target for therapeutic intervention (5).

In humans, IAV causes annual epidemics of respiratory illness with an estimated annual mortality rate of 290,000 to 600,000 worldwide (6). Sporadic pandemics, associated with antigenically novel IAV strains, can lead to increased morbidity and mortality compared to seasonal epidemics (7). Secondary bacterial coinfection is the most common complication of primary IAV, and a high incidence is reported during both epidemics and pandemics. The two bacterial species most commonly isolated from IAV coinfection patients are the Gram-positive species Streptococcus pneumoniae and Staphylococcus aureus (8). During the most devastating human IAV pandemic, the 1918 H1N1 subtype “Spanish flu,” bacterial coinfection was identified in 70% to 90% of autopsies (7). In the most recent pandemic of 2009, 25% to 40% of mortalities were attributed to bacterial coinfection, despite the widespread use of antibiotics (9, 10).

S. aureus is found as a commensal organism in around 25% to 40% of the healthy human population but is responsible for an array of diseases, ranging from uncomplicated skin and soft tissue infections to life-threatening conditions, such as endocarditis and necrotizing pneumonia (11). Of note, the emergence of highly virulent clones of community-associated methicillin-resistant S. aureus (CA-MRSA) in recent years has resulted in S. aureus becoming the leading cause of nosocomial pneumonia in the United States (12). The role of immune dysregulation during coinfection has been extensively studied, and it is believed to be one of the main underlying causes for the increased susceptibility to bacterial coinfection following primary influenza (8). The contribution of individual S. aureus factors is less known. *In vivo*, the SaeR/S system has been shown to contribute to coinfection in a murine model (13). *In vitro*, phenol-soluble modulins have been demonstrated to be more cytotoxic in lung epithelial cells (ECs) previously infected with IAV (14), and incubation with IAV resulted in virus bound to bacterial cells and increased adherence of S. aureus to epithelial cells (15). Additionally, Tashiro and colleagues (16, 17) reported that S. aureus strain Wood 46 secretes a protease which can substitute for (or augment) the host proteases required to activate IAV HA, thereby enhancing the production of infectious viral particles. However, the identity of this protein remained unknown (16, 17), hindering further investigation into possible intervention mechanisms.

In the current study, we investigated the ability of secreted proteins of S. aureus to enhance IAV replication *in vitro*. Unexpectedly, we discovered that a single polypeptide, lipase 1, potentiates IAV replication *in vitro* and *in vivo*, independently of all known S. aureus proteases. Lipase 1 acts during the late stages of IAV replication, separately from HA cleavage, leading to an increase in the number of infectious particles produced. These findings expand on our understanding of the molecular events that occur during IAV-S. aureus coinfection and identify a novel role for one of the most abundant S. aureus-secreted proteins (18).

## RESULTS

### S. aureus proviral activity is mediated by the lipolytic activity of lipase 1.

**FIG 1:**
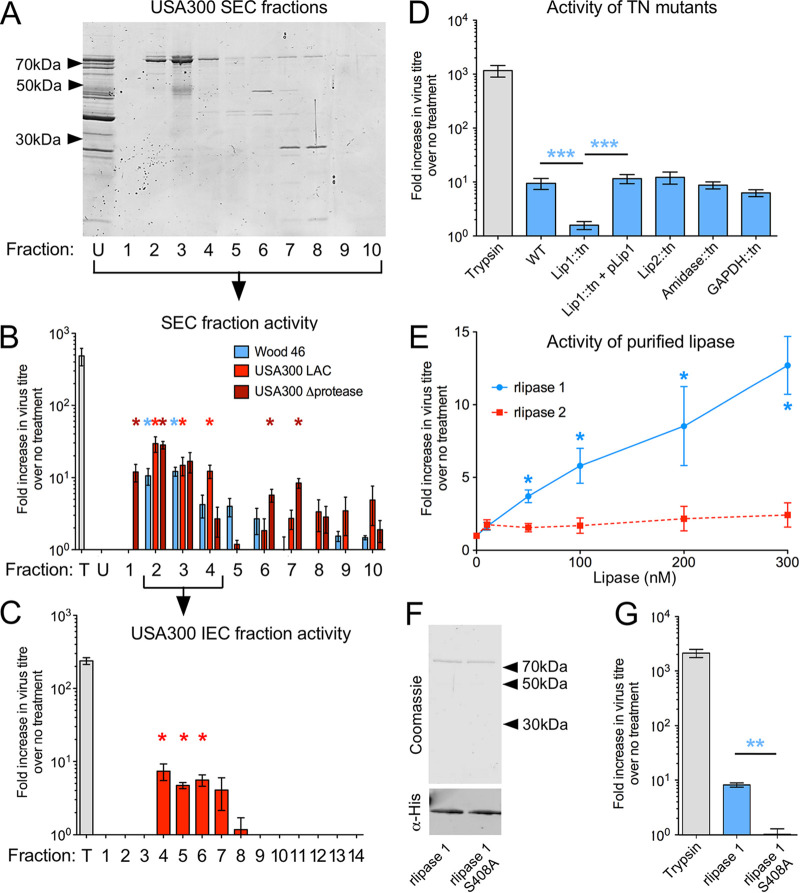
S. aureus lipase 1 is proviral for IAV *in vitro*. (A) Supernatants from S. aureus cultures were separated by SEC and 10-μl volumes of the first 10 fractions separated by SDS-PAGE and stained for total protein. Representative images of USA300 LAC fractions are shown. U, unfractionated. (B) CEF cells were infected with PR8 at an MOI of 0.01 and, immediately postabsorption, treated with trypsin (T; 2.5 μg/ml) or aliquots of unfractionated or fractionated supernatants from the indicated S. aureus strains. Virus titers were determined by plaque assay at 48 hpi and normalized to the level of an untreated control. (C) SEC fractions 2 to 4 from WT USA300 LAC were pooled and separated by IEC before testing for pro-IAV activity was performed as described above. Data shown are means ± standard errors of the means of results from 3 independent infections. (D) Cells of transposon (tn) insertion mutants of indicated genes or a plasmid-complemented (Lip1::tn + pLip1) strain were grown and supernatants separated by SEC before testing for proviral activity was performed. Fractions were tested separately, but data shown represent the average values of results from fractions 1 to 5. (E) Increasing concentrations of purified rlipase 1 or rlipase 2 were added to CEF cells infected with PR8 at MOI 0.01. Infections were harvested after 48 h and infectious titers determined. (F) Aliquots of purified recombinant wild-type or catalytic site mutant (S408) lipase 1 were analyzed by SDS-PAGE and staining with Coomassie blue or Western blotting with anti-histidine IgG. (G) A final concentration of 300 nM lipase 1 or lipase 1 S408A was added to CEF cells infected with PR8 at MOI 0.01. Infections were harvested after 48 h and infectious titers determined. Numerical data shown are means ± standard errors of the means of results from 3 independent protein preparations (B, D, E, and G). Single asterisks (*), double asterisks (**), and triple asterisks (***) indicate *P* values of <0.05, <0.01, and <0.001, respectively, based on Student’s *t* test, compared to virus-only controls (B, C, and E), Tn-Lip1 (D and E), and rlipase 1 (F and G) results.

A previous study by Tashiro et al. reported that activity of an S. aureus-secreted protease enhanced IAV replication via HA cleavage (16, 17). However, S. aureus produces a wide array of proteins involved in pathogenesis, including 10 secreted proteases (19). To investigate further the role of S. aureus proteases during influenza coinfection, we repeated the protocol employed by Tashiro et al. and fractionated culture supernatants of S. aureus strains Wood 46 (Fig. 1A), USA300 LAC and a deletion mutant of USA300 LAC deficient in the production of all known secreted proteases (20) (see Fig. S1A and B in the supplemental material) by size exclusion chromatography (SEC). Aliquots of the resulting SEC fractions were added to primary chicken embryo fibroblast (CEF) cells infected with the H1N1 IAV strain A/Puerto Rico/8/34 (PR8) to test for their ability to support virus replication in the absence of exogenous trypsin. As expected, the addition of trypsin increased virus titer several-hundred-fold compared to samples with no exogenous protease (Fig. 1B). Fractions 2 to 4 from both Wood 46 and USA300 LAC culture supernatants also showed significantly increased virus replication (Fig. 1B), consistent with the original report of Tashiro et al. (17). However, unexpectedly, supernatant fractions from the protease-deficient USA300 LAC strain retained proviral activity equivalent to that of the wild-type (WT) fractions (Fig. 1B), indicating that the pro-IAV activity was independent of the presence of known secreted proteases.

10.1128/mBio.00975-20.1 FIG S1 S. aureus proviral activity is independent of known secreted proteases. (A and B) Stationary-phase supernatant from Wood 46 (A) or USA300 LAC Δprotease (B) was separated by SEC, and 10-μl volumes of the first 10 fractions were separated by SDS-PAGE and stained for total protein. (C) SEC fractions 2 to 4 from WT USA300 LAC were pooled and separated by cation exchange chromatography. All fractions (10 μl) were separated by SDS-PAGE and silver stained. U, unfractionated; RT, column flowthrough, W, wash. Images representative of multiple independent purifications are shown. Download FIG S1, TIF file, 2.8 MB. Copyright © 2020 Goncheva et al. 2020 Goncheva et al. This content is distributed under the terms of the Creative Commons Attribution 4.0 International license.

To identify the bacterial factor responsible for the observed proviral effect, the S. aureus SEC fractions were further separated by ion-exchange chromatography (IEC; Fig. S1C), after which 3 samples were found to have consistently retained proviral activity (Fig. 1C). These were analyzed by tryptic digestion followed by liquid chromatography-tandem mass spectrometry (LC-MS/MS), and two proteins, lipase 1 (*gehA*) and lipase 2 (*gehB*), were found to be common to the active fractions (see Table S1 in the supplemental material). The proviral activity of these and of two other high-scoring mass spectrometry candidates, N-acetylmuramoyl-l-alanine amidase domain-containing protein (amidase) and glyceraldehyde-3-phosphate dehydrogenase (GAPDH) were tested by utilizing the Nebraska transposon (Tn) mutant library, constructed in the USA300 LAC background (21). Culture supernatants from the relevant Tn insertion mutants were fractionated by SEC and tested for their ability to promote IAV replication as described above. Disruption of the lipase 2, amidase, or GAPDH genes had no impact on the proviral activity of USA300 culture supernatants (Fig. 1D). In contrast, disruption of the lipase 1 gene reduced the stimulatory activity to background levels. Furthermore, the proviral phenotype could be restored by complementation of the lipase 1 Tn mutant with a plasmid encoding lipase 1 (pLip1; Fig. 1D). Taken together, these data demonstrate that the observed proviral activity of S. aureus USA300 LAC is dependent on the presence of lipase 1.

10.1128/mBio.00975-20.7 TABLE S1 Identities of proteins present in proviral fractions. Data represent the proteins identified in four proviral fractions of S. aureus supernatant by LC-MS/MS. Hits with an overall score of >40 and with more than 2 peptides are shown. Download Table S1, DOCX file, 0.02 MB. Copyright © 2020 Goncheva et al. 2020 Goncheva et al. This content is distributed under the terms of the Creative Commons Attribution 4.0 International license.

To further characterize the effect of S. aureus lipase 1 on IAV replication, we produced polyhistidine-tagged recombinant forms of both lipase 1 (rlipase 1) and the paralog lipase 2 (rlipase 2) in Escherichia coli and purified them by immobilized metal affinity chromatography (IMAC) (Fig. S2A and B). Both protein preparations exhibited concentration-dependent lipolytic activity *in vitro*, with lipase 2 showing higher activity than lipase 1 (Fig. S2C and D), consistent with previous reports (5, 18, 22). When CEF cells infected with IAV PR8 were incubated with the recombinant S. aureus lipase 1 (rlipase 1), there was a concentration-dependent increase in IAV titer, whereas recombinant lipase 2 (rlipase 2) had no proviral effect (Fig. 1E). The lipolytic activity of lipase 1 has been mapped to serine 408 and histidine 643 (5), so to test if the proviral phenotype of the protein was due to lipase activity, a site-directed mutant form of rlipase 1 (rlipase 1 S408A), with the serine replaced by alanine, was similarly produced in E. coli and purified (Fig. 1F). Importantly, mutation of the active site of rlipase 1 dramatically reduced both the lipolytic activity (Fig. S2E) and the proviral activity (Fig. 1G) of the protein, suggesting that the lipase enzymatic activity was responsible for the proviral effect.

10.1128/mBio.00975-20.2 FIG S2 Recombinant lipase 1 and lipase 2 are lipolytic enzymes. (A) 6xHistidine-tagged lipase 1 was purified by native immobilized metal affinity chromatography (IMAC). All fractions were diluted 1:10, separated by SDS-PAGE, and stained for total protein (left) or were transferred to a membrane followed by Western blotting performed with an antihistidine antibody (right). Fraction 4 was chosen and used for further work. (B) 6xHistidine-tagged lipase 2 was purified by native IMAC. All fractions were diluted 1:10, separated by SDS-PAGE, and stained for total protein (left) or transferred to a membrane followed by Western blotting performed with an anti-histidine-tagged antibody (right). Fraction 4 was chosen and used for further work. (C and D) A range of final concentrations (indicated in nanomolar) of rlipase 1 (C) or rlipase 2 (D) were used in a Tween-based lipase assay. The assay was performed in a plate reader, at 37°C, with optical density (OD) readings taken at 495 nm every 5 min for 18 h. Data shown are means ± standard errors of the means of results from 3 independent protein preparations. (E) 6xHistidine-tagged lipase 1 or lipase 1 containing a S408A substitution was purified by native IMAC, and a final concentration of 300 nM of rlipase 1 or rlipase 1 S408A was used in a Tween-based lipolytic assay as described for panel C. Data shown are means ± standard errors of the means of results from 3 independent protein preparations. Download FIG S2, TIF file, 2.7 MB. Copyright © 2020 Goncheva et al. 2020 Goncheva et al. This content is distributed under the terms of the Creative Commons Attribution 4.0 International license.

### S. aureus lipase 1 acts during a single IAV replication cycle.

**FIG 2:**
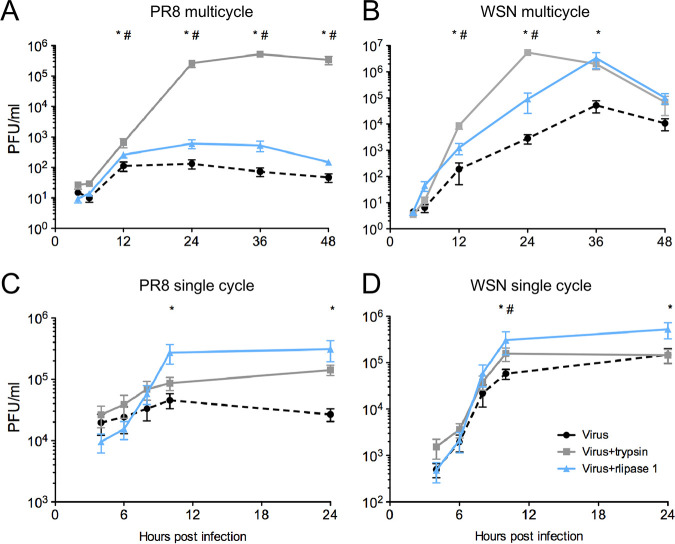
Lipase 1 increases viral replication during a single infectious cycle. CEF cells were infected with PR8 (A) or WSN (B) at an MOI of 0.01 or with PR8 (C) or WSN (D) at an MOI of 3 in multicycle experiments (A and B) or single-cycle experiments (C and D). PBS or a final concentration of 300 nM rlipase 1 or 2.5 μg/ml trypsin was added immediately postinoculum removal. Infectious titers were determined at the indicated times. Data shown are means ± standard errors of the means of results from 4 independent recombinant protein preparations. A single asterisk (*) indicates a *P* value of <0.05 for results of comparisons between the virus group without lipase 1 and the virus group with lipase 1, and a crosshatch symbol (#) indicates a *P* value of <0.05 for results of comparisons between the virus group without trypsin and the virus group with trypsin, based on Student's *t* test.

To examine further the rlipase 1 effect on IAV, growth of the virus in the presence of rlipase 1 was investigated in detail. IAV requires specific proteolytic cleavage of HA to produce infectious virus capable of initiating a new cycle of infection. *In vitro*, this is normally mediated by exogenously added trypsin. As expected, infection of CEFs at a low multiplicity of infection (MOI) with trypsin supported at least two successful rounds of infection (Fig. 2A, gray squares). Infection at low MOI with rlipase 1, in the absence of exogenous protease, resulted in enhanced IAV replication during the first replication cycle but thereafter had little effect (Fig. 2A, blue triangles). However, when the partially trypsin-independent virus A/WSN/33 (WSN) (23, 24) was utilized, rlipase 1 increased virus yield across the whole time course, with true multicycle replication kinetics (Fig. 2B). Growth under high-MOI conditions similarly demonstrated that the presence of rlipase 1 increased IAV titer during a single round of replication for both strains of IAV (Fig. 2C and D). Thus, rlipase 1 enhanced replication of a trypsin-dependent IAV in a single replication cycle but did not support multiple rounds of infection, suggesting that it was not acting as if it were mediating proteolytic cleavage of HA.

### Lipase 1 is broadly proviral for IAV.

Lipase 1 has been reported to be one of the most abundantly secreted factors during the stationary phase of growth in the USA300 LAC strain (18). However, the distribution and level of expression of lipase 1 among S. aureus strains are poorly understood. Examination of 8,334 publicly available S. aureus genomes identified the lipase 1 gene (*gehA*) in 8,274 (99.3%), indicating broad conservation across the species (data not shown). Furthermore, Western blotting of culture supernatants from 19 clinical isolates using an antibody specific for lipase 1 detected expression in 16 (84%) isolates under nutrient-rich *in vitro* conditions (Fig. S3), indicating that lipase 1 is broadly expressed by S. aureus strains.

10.1128/mBio.00975-20.3 FIG S3 Lipase 1 expression can be detected across a range of S. aureus isolates. S. aureus strains representing a number of important sequence types (ST), or from confirmed IAV-S. aureus coinfections (Table S1), were selected and the levels of production of lipase 1 investigated. The indicated strains were grown O/N to stationary phase in TSB in the absence of IAV and normalized to an OD_600_ of 3.0, and the supernatants were harvested. Supernatants (or a final concentration of 200 nM rlipase 1 as a marker; M) were separated by SDS-PAGE and stained for total protein (top) or were transferred to a membrane followed by Western blotting performed with anti-lipase 1 antibody (bottom). Lipase 1 is indicated with a red arrow. A large amount of nonspecific background signal is present on the Western blot due to the endogenous production of protein A by S. aureus, which binds IgG nonspecifically (blue arrows). Note that different strains often produce differing profiles of secreted proteins, which accounts for the diversity of expression profiles observed on the SDS-PAGE gel. Signs under the images indicate whether the strain was deemed to express lipase 1 (+) or not (-). Download FIG S3, TIF file, 1.9 MB. Copyright © 2020 Goncheva et al. 2020 Goncheva et al. This content is distributed under the terms of the Creative Commons Attribution 4.0 International license.

**FIG 3:**
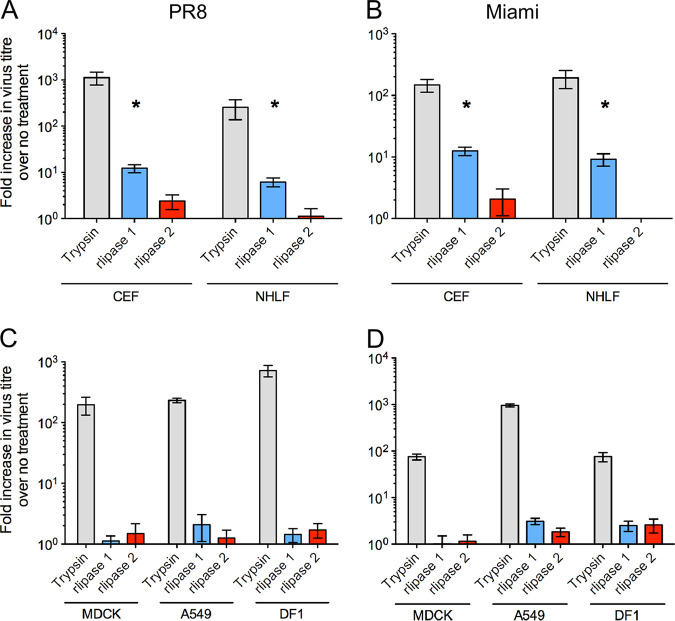
Lipase 1 proviral activity is restricted to primary cells. The indicated cell lines were infected with PR8 (A and C) or Miami (B and D) IAV at an MOI of 0.01 and a final concentration of 300 nM rlipase 1 or rlipase 2 or of 2.5 μg/ml trypsin was added. Infectious titers were determined after 48 h. All data shown are means ± standard errors of the means of results from 3 independent protein preparations. A single asterisk (*) indicates a *P* value of <0.05, based on a Student's *t* test, compared to the virus-only control.

To investigate if rlipase 1 had proviral activity for other strains of IAV and if this could extend to human cells, infections were performed using the H3N8 A/equine/Miami/63 (Miami) strain and primary normal human lung fibroblast (NHLF) cells. Importantly, rlipase 1 enhanced replication of PR8 and Miami in both CEF and NHLF cells, respectively, but rlipase 2 did not (Fig. 3A and B). To determine the breadth of the proviral activity of rlipase 1, we tested a range of IAV strains in CEF and NHLF cells, under both single-cycle and multicycle growth conditions. Proviral activity was observed for all viruses tested, including avian strains, and for both cell types (Table 1). Taken together, these data demonstrate that rlipase 1 has broad proviral activity for IAV in human cells. We also performed infections in relevant primary and continuous cell lines, including Madin Darby canine kidney (MDCK) cells (used for IAV quantification by plaque assay), A549 human lung epithelial cells, DF1 cells (a spontaneous immortalized derivative of primary CEF cells), and primary human bronchial-tracheal epithelial cells (HBTECs). Of note, we did not observe a rlipase 1-mediated increase in viral titer in any of these cell lines (Fig. 3C and D) or in HBTECs (Fig. S4). Taken together, these data suggest that the proviral phenotype of S. aureus rlipase 1 may be specific for cells that are primary and that are of fibroblast origin.

**TABLE 1:** Lipase 1 is proviral for a range of mammalian and avian viruses in primary human and avian fibroblasts^*a*^

Virus	Host	Subtype	Avg fold titer increase (±SD)			
			CEF cells		NHLF cells	
			MOI 0.01	MOI 3	MOI 0.01	MOI 3
A/Puerto Rico/8/34	Human	H1N1	14.2 (3.2)	11.4 (4.1)	5.72 (2.7)	4.69 (2.9)
A/Equine/Miami/63	Horse	H3N8	12.6 (3.3)	9.85 (5.1)	7.50 (4.7)	2.32 (0.9)
A/Udorn/307/72	Human	H3N2	14.7 (5.3)	10.4 (0.78)	5.73 (2.2)	3.60 (1.9)
A/Duck/England/62	Duck	H4N6	207.2 (88.8)	13.8 (6.1)	6.94 (3.2)	11.25 (5.4)
A/Turkey/Canada/63	Turkey	H3N2	57.7 (32.6)	64.6 (38.5)	Not tested	Not tested
A/Mallard/Netherlands/10/99	Duck	H1N1	54.4 (29.1)	15.5 (6.1)	Not tested	Not tested

a CEF or NHLF cells were infected with the indicated viruses at a MOI of 0.01 or 3, and 300 nM rlipase 1 was added immediately after inoculum removal. Samples were harvested at 24 h (MOI of 3) or 48 h (MOI of 0.01) and infectious viral titers determined by plaque assay. Data are expressed as the average (± standard deviation) fold increase in titer compared to parallel samples incubated without trypsin and represent results from 3 independent experiments.

10.1128/mBio.00975-20.4 FIG S4 Lipase 1 does not exhibit a proviral effect during infection of primary human bronchial/tracheal epithelial cells. Monolayers of primary human bronchial/tracheal epithelial cells were infected with PR8 at a MOI of 0.01 and treated with 2.5 μg/ml trypsin, a final concentration of 300 nM rlipase 1, or 300 nM rlipase 1S408A. Supernatant was harvested at 48 hpi, and infectious viral titers were determined by plaque assay. Data shown represent fold increases in virus-only samples. Data shown are means ± standard errors of the means of results from 2 experiments, with 2 technical replicates per experiment. Download FIG S4, TIF file, 0.5 MB. Copyright © 2020 Goncheva et al. 2020 Goncheva et al. This content is distributed under the terms of the Creative Commons Attribution 4.0 International license.

### Lipase 1 exerts proviral activity during the late stages of IAV replication.

**FIG 4:**
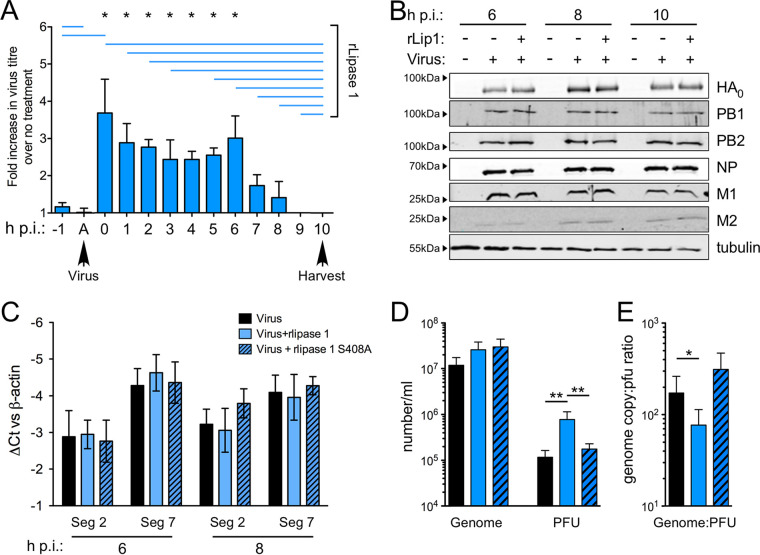
Lipase 1 increases the ratio of infectious particles produced during an infection. (A) CEF cells were treated with a final concentration of 300 nM rlipase 1 for the periods indicated by the blue lines before or after being infected with PR8 at MOI of 1. Lane A, a 1-h virus adsorption step was performed at 37°C followed by an acid wash step following inoculum removal to destroy uninternalized virus. All samples were harvested at 10 hpi and titrated by plaque assay. Data shown as means ± standard errors of the means of data representing fold change with respect to the virus-only sample for 5 independent recombinant protein preparations. (B) CEF cells infected with PR8 at MOI of 1 were lysed and Western blotting was performed as indicated. (C) CEF cells were infected as described for panel B and lysed at the indicated time points, and total RNA was extracted. qRT-PCR was performed for PR8 segment 2 (Seg 2) and Seg 7 RNAs, and values were normalized to chicken actin transcripts. Data shown are means ± standard errors of the means of results from 2 independent protein preparations and primary cell isolations. (D and E) CEF cells were infected as described for panel B, and cell lysate was used for extraction of RNA and assay of virus titers at the indicated times. Copies of viral genomic RNA from segments 2 and 7 were quantitated by qRT-PCR (D), and genome copy number-to-PFU ratios were calculated (E). Data shown are means ± standard errors of the means of results from 3 independent protein preparations and primary cell isolations. Single asterisks (*) and double asterisks (**) indicate *P* values of <0.05 and <0.01, respectively, as assessed by one-way ANOVA and Dunn’s multiple-comparison test.

We considered that the increase in the number of infectious particles seen during a single infectious cycle might have been due to (i) increased levels of attachment and entry, (ii) more-efficient production of virus components, or (iii) improved assembly and release from the cell. To investigate this, rlipase 1 was added at different stages of a single-cycle infection (Fig. 4A). The presence of rlipase 1 before infection or during virus absorption did not lead to an increase in titer. However, addition of rlipase 1 after the absorption period at any time up to the first 6 h postinfection (hpi) resulted in significant increases in titer, whereas addition at 7 h onward had no significant influence. These data suggested that rlipase 1 was not affecting the attachment and/or internalization of the virus but was acting later in the replication cycle. It is also possible that rlipase 1 affects virus replication in both early and late events, a scenario that this experimental setup could not exclude. To determine if rlipase 1 induced increased production of viral components, we analyzed the accumulation of viral proteins at different time points. No differences were observed between virus-infected cells and infected cells treated with rlipase 1 (Fig. 4B). Furthermore, analysis of intracellular viral RNA also showed no significant differences between treated and untreated cells in the levels of accumulation of viral segment 2 or viral segment 7 RNAs produced (Fig. 4C). Therefore, rlipase 1 did not seem to be affecting viral macromolecular synthesis, thus suggesting an effect on assembly and/or release. To investigate the final steps of viral replication, we quantified the amounts of viral genome (a measure of overall virus particle formation) and infectious particles released into the culture supernatant. The addition of either active rlipase 1 or the catalytically inactive S408A mutant had no significant effect on the amounts of viral genomic RNA released at 8 hpi, but as before, the presence of active rlipase 1 but not inactive rlipase 1 significantly increased the titer of infectious virus (Fig. 4D). Consequently, this caused a significant decrease in the genome copy number/PFU ratio of the virus population (Fig. 4E). Similar effects were seen when earlier (6 hpi) or later (10 hpi) time points were analyzed (Fig. S5A and B), indicating that rlipase 1 increased the infectivity of released virus. The original analysis of the molecular basis of S. aureus enhancement of IAV disease postulated that a bacterial protease was responsible for cleaving viral HA (16, 17). To determine if lipase 1 was able to cleave HA, partially purified virus preparations with either uncleaved or cleaved HA were treated with either rlipase 1 or trypsin. However, no HA cleavage was detected in the presence of rlipase 1 (Fig. S5C).

10.1128/mBio.00975-20.5 FIG S5 Lipase 1 increases the ratio of infectious particles produced during an infection but not by cleaving HA. (A and B) Supernatants from CEF cells infected with PR8 at a MOI of 1 were collected at the indicated times, and titers were determined for copies of viral genomic RNA from segments 2 and 7 by qRT-PCR and infectious titer by plaque assay. (B) Genome copy number-to-PFU ratios were calculated. Data shown are means ± standard errors of the means of results from 3 independent protein preparations and primary cell isolations. An asterisk (*) indicates a *P* value of <0.05, assessed by one-way ANOVA and Dunn’s multiple-comparison test. (C) Partially purified virus with uncleaved HA (left) or cleaved HA (right) was treated with PBS (-), 2.5 μg/ml trypsin (T), or 300 nM rlipase 1 (L) for 1 h at 37°C. Samples were then separated on a 12% SDS-PAGE gel, and Western blotting was performed with goat polyclonal anti-whole H1N1 virus antibody. The images shown are representative of the results. Download FIG S5, TIF file, 2.0 MB. Copyright © 2020 Goncheva et al. 2020 Goncheva et al. This content is distributed under the terms of the Creative Commons Attribution 4.0 International license.

**FIG 5:**
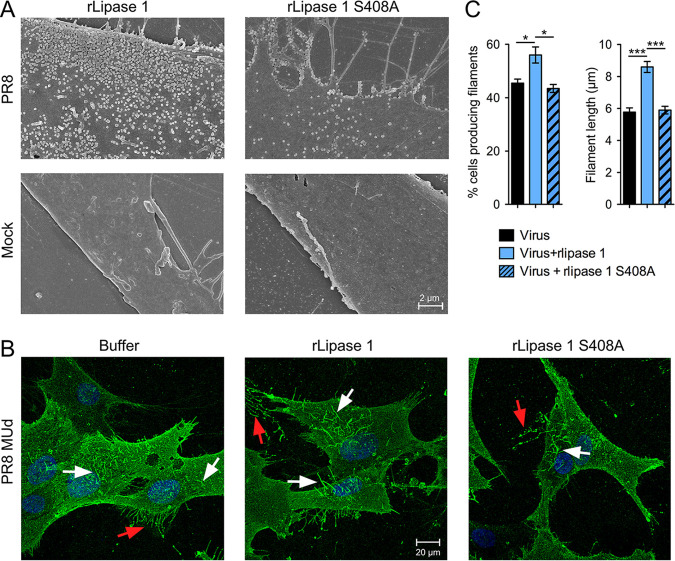
Lipase 1 positively modulates virus budding. (A) CEF cells were infected with PR8 at an MOI of 3 and treated with a final concentration of 300 nM active or inactive (S408A) rlipase 1, and at 8 hpi, cells were fixed and processed for SEM. Representative images are shown at magnification of ×10,000. The scale bar indicates 2 μm. (B and C) CEF cells were infected with PR8 MUd at MOI of 3 treated with rlipase 1 or buffer as described above, and cells were fixed at 8 hpi and subjected to surface staining with an anti-PR8 antibody. Cells were imaged on a Leica confocal microscope. White arrows indicate viral filaments, and red arrows indicate cellular retraction fibers. Scale bar, 20 μm. (C) Images from panel B were analyzed, and the number of filament-producing cells and the filament length were determined. A minimum of 60 cells were counted and a minimum of 60 filaments measured from 3 independent experiments. A single asterisk (*) indicates a *P* value of <0.05, and triple asterisks (***) indicate a *P* value of <0.001, based on a one-way ANOVA.

The finding that the proviral effect of lipase 1 depended on its enzymatic activity suggested the hypothesis that it might affect the process of virus budding through the plasma membrane. Consistent with this, analysis of the cell surface by scanning electron microscopy (SEM) showed that, at 8 h after infection with PR8, there were notably more virus particles budding from cells treated with active rlipase 1 than with from those treated with the catalytically inert version of the protein (Fig. 5A). The PR8 strain of IAV produces only spherical virus particles, but most human clinical strains of IAV also produce micrometer-length filamentous particles (25). To examine the cells for effects on this form of virus budding, we utilized a filamentous derivative of PR8 (PR8 MUd) containing segment 7 from the filamentous virus A/Udorn/307/1972 (26, 27). Treatment of PR8 MUd-infected CEF cells with active rlipase 1 but not inactive rlipase 1 resulted in a significant increase in the number of infected cells producing viral filaments, as well as in the length of the filaments (Fig. 5B and C). Thus, overall, the results showed that rlipase 1 acts late in the viral life cycle to favorably modulate IAV morphogenesis.

### Lipase 1 can enhance IAV vaccine production *in ovo*.

**FIG 6:**
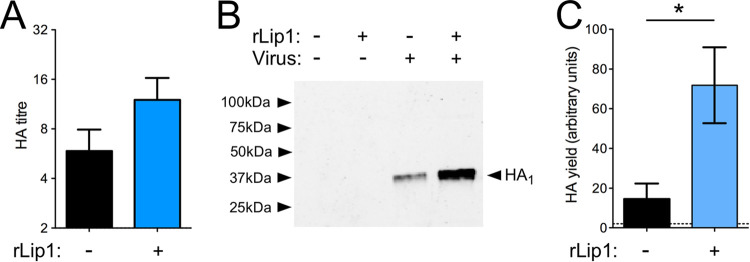
Lipase 1 enhances IAV replication *in ovo*. (A) Groups of 4 to 6 10-day-old embryonated eggs were infected with 100 PFU of a Cal07:PR8 6:2 reassortant virus (pH1N1), and a final concentration of 100 nM rlipase 1 or 50 mM Tris was added to the virus. Eggs were chilled 48 h later, and allantoic fluid was harvested and HA titer determined. (B) Samples from the experiment described in panel A were pooled, virus was partially purified through a sucrose cushion, and HA was deglycosylated prior to Western blotting for HA. Representative images are shown. (C) The signal intensity of Western blot bands was quantified. Data shown are from 3 independent experiments performed with separate protein preparations, including 4 to 6 eggs per group per experiment. A single asterisk (*) indicates a *P* value of <0.05, based on paired Student's *t* tests.

In order to examine the effect of lipase 1 *in vivo*, we initially utilized a murine model of IAV-S. aureus coinfection where bacteria were introduced 1 day after IAV infection, but there were no significant differences in weight loss or clinical scores and we saw only a moderate increase in viral titer in the coinfected animals, regardless of the presence or absence of lipase 1 (Fig. S6). In addition, complete bacterial clearance had occurred by day 2 postcoinfection (data not shown). Animal models of IAV-S. aureus coinfection are limited in their capacity to recapitulate the conditions of human respiratory infection (8). Furthermore, a number of studies have indicated that conventional mouse models have major limitations for the study of S. aureus, due to immune system activation or receptor incompatibility for S. aureus effector proteins (28, 29). Accordingly, we next employed another established *in vivo* system of IAV replication—embryonated hen’s eggs. Importantly, this system is also used for the commercial production of IAV vaccine, so we used a reassortant virus with glycoprotein genes from the 2009 H1N1 pandemic isolate A/California/07/2009 and the remaining segments from PR8 to mimic a vaccine strain of IAV (30). Addition of ∼100 nM rlipase 1 to 10-day-old embryonated eggs did not result in toxicity to the embryos (data not shown). Addition of rlipase 1 to eggs infected with the reassortant virus resulted in increased average HA titers compared to virus-only samples, although the results did not reach statistical significance (Fig. 6A). However, an assessment of the amounts of HA_1_ in partially purified virus preparations by Western blotting following deglycosylation (30) showed that the addition of rlipase 1 greatly improved the yield of the vaccine antigen (Fig. 6B). Quantification of HA_1_ from replicate experiments revealed a 5-fold increase in protein yield following treatment with rlipase 1 (Fig. 6C). Thus, rlipase 1 enhanced IAV replication *in vivo*, in a manner similar to that observed *in vitro*, regardless of the presence of proteases *in ovo* that cleave IAV HA and facilitate multicycle infection. Importantly, these findings suggest a potential application of rlipase 1 activity for the enhancement of IAV vaccine yield, which is currently a major limitation of standard methods for the production of influenza vaccines.

10.1128/mBio.00975-20.6 FIG S6 Murine coinfection with IAV and S. aureus does not lead to an increase in viral titer. Groups of 5 female BALB/c animals were infected with PBS or 10 PFU PR8 intranasally in a mixture with 40 μl PBS on day 0. At 24 h later, animals were further challenged with 40 μl of 1 × 10^7^ CFU of WT or lip1::tn S. aureus or PBS intranasally. (A) Weight loss postinfection. (B) Daily clinical scores of 0 to 3 were assigned for inactivity, ruffled fur, and labored breathing. A cumulative score representing the scores determined for all criteria is presented for each animal. (C) At 48 h after bacterial challenge, lungs were harvested and viral titers were determined by standard plaque assay. No bacteria were recovered from any of the animals. All data shown represent means ± standard errors of the means of results. Download FIG S6, TIF file, 1.2 MB. Copyright © 2020 Goncheva et al. 2020 Goncheva et al. This content is distributed under the terms of the Creative Commons Attribution 4.0 International license.

## DISCUSSION

Secondary S. aureus infection is a major cause of morbidity and mortality in patients with influenza. A previous report indicated that an unidentified protease from S. aureus strain Wood 46 was responsible for enhanced IAV replication in primary avian cells (17). Here, we demonstrated that S. aureus lipase 1 enhances IAV replication during infection of primary human and avian cells *ex vivo* and *in ovo* and that this activity is independent of known secreted proteases. Instead, our data indicate that S. aureus lipase 1 is responsible for this proviral effect and that this applies to a broad array of IAV subtypes of mammalian and avian origin (Table 1). Lipase 1 is one of the most abundantly expressed proteins secreted by the USA300 S. aureus strain (18), but an understanding of its biological role is lacking. *In vivo* expression has been implied due to detection of circulating anti-lipase antibodies (31, 32), and lipolytic activity on short-chain triglycerides has been reported, although the kinetics data suggest that it has lower activity than lipase 2 (18, 22, 33, 34). The specific lipolytic activity of lipase 2 has also been shown to prevent innate immune cell activation by inactivating bacterial lipoproteins and thus blocking recognition by macrophages (35). However, to date, no role for lipase 1 in the pathogenesis of S. aureus has been identified. Here, we provide the first report of a role for lipase 1 activity during coinfection with IAV.

Our data indicate that the effect of lipase 1 on IAV occurs during the late stages of virus replication. We propose that lipase 1 acts by modifying host cell lipids in the cell plasma membrane, leading to more efficient budding and to production of an increased number of infectious IAV particles per replication cycle. Although understanding of IAV assembly and particle budding is incomplete (36), membrane modulation is required for the formation and release of new particles, and specific lipid structures, in and out of lipid rafts (37–40), have been implicated as preferred budding sites. Conversely, previous work has indicated that budding of the virus can be altered both positively and negatively by perturbation of membrane composition (41). The lipase 1-mediated phenotype was observed only in primary fibroblast cells, and it is possible that the lipids modified by lipase 1 are not present or are differentially regulated in immortalized cells. Indeed, immortalized cells have been reported to have numerous attenuations affecting innate intracellular immunity (42) and the lipid composition of membranes (43). This is consistent with the differences in proviral activity between lipase 1 and lipase 2, which have been shown to have different substrate preferences (5, 18). The apparent specificity of lipase 1 for fibroblast cells is noteworthy, as these cells are present in most lung spaces and account for about 10 to 20% of all lung cells (44, 45) and have been demonstrated to be recruited during IAV infection (46). Furthermore, the effect of lipase 1 on fibroblasts could partially reflect the specific timing of bacterial coinfection. Coinfection by S. aureus normally occurs around day 7 of IAV infection, after the viral peak (47), at a time when the lung environment begins tissue repair. Lung repair is spearheaded by fibroblasts, and these cells are recruited heavily to sites of virus-induced damage (48). As such, the increase in levels of available target cells may exacerbate the effect of lipase 1 during coinfection, resulting in the resurgence of viral titer observed during coinfection (8, 47).

The proviral activity of rlipase 1 for NHLF cells *ex vivo* further suggests a potential role during human clinical infection, and the enhanced virus replication *in ovo* indicates the relevance of the lipase 1 activity for IAV in a complex *in vivo* environment. Animal models have demonstrated disease exacerbation upon coinfection (47), and expression of lipase 1, which is widespread in S. aureus (see Fig. S4 in the supplemental material), has been suggested to occur during human infection, based on the detection of antibodies (31, 32). Accordingly, we suggest that S. aureus superinfection of humans with influenza may lead to a lipase 1-mediated enhancement of virus replication, resulting in a more prolonged and severe infection. Furthermore, the damage caused by the increased IAV replication in fibroblasts in the lung may lead to increased occurrence of fibrosis, which has been demonstrated during bacterial coinfection (10). Additionally, IAV-S. pneumoniae coinfection models have demonstrated the viral neuraminidase can cleave sialic acid (49), which bacteria can utilize as an energy source (50), and IAV infection also results in an increase in levels of host adhesion molecules, such as fibronectin (51), that both S. pneumoniae and S. aureus can bind. Therefore, enhanced virus replication could facilitate the spread and replication of bacteria, suggesting an indirect beneficial role for lipase 1 in the pathogenesis of S. aureus during coinfection. Although it is well established that secondary bacterial pneumonia is a major cause of mortality during IAV epidemics, animal models of IAV-S. aureus infection have offered differing pictures of the outcome of coinfection. While some murine models have demonstrated increased severity of S. aureus respiratory disease when preceded by IAV infection (52), in a cynomolgus macaque model, prior infection with IAV did not predispose the animals to more-severe infection with S. aureus USA300 (53). The authors concluded that the distinct observations made compared to human clinical and rodent model data may have been due to variation in the strain of virus employed or to a host species-specific effect on susceptibility to IAV or S. aureus infection. Alternatively, it was suggested that the findings may indicate that unknown comorbidities are required to promote the synergistic effect of IAV-S. aureus coinfection (53).

Although we did not see an effect of lipase 1 in the murine coinfection model employed in the current study, this may have been due to the timing of bacterial challenge relative to IAV infection, prior to the maximal recruitment of fibroblasts, or to general limitations of the murine model for replicating human respiratory infection as previously established (8, 28, 29). Furthermore, the bacteria were rapidly cleared from the lungs and levels of lipase 1 expression may not have been sufficient to mediate a proviral effect. However, using an established embryonated egg model, a clear proviral effect for rlipase 1 was identified *in vivo*. This observation suggests that a potential application of the current finding is the utilization of rlipase 1 as a growth enhancer for IAV vaccine production. Vaccine production can be inefficient, particularly with recent H1N1 and H3N2 isolates, which grow poorly in eggs due to receptor incompatibilities (54, 55). The use of rlipase 1 could significantly enhance the yield of poorly growing viruses, as demonstrated here with the pandemic 2009 H1N1 reassortant virus (Fig. 6). Of note, *in vitro* data obtained from studies performed with CEF cells indicated that rlipase 1 was active on avian strains of IAV (Table 1), which could be a considerable advantage in the event of an influenza pandemic caused by a strain bearing an avian strain-derived HA (56).

The threat of another global influenza pandemic is ongoing, and bacterial coinfection is a frequent and major complication of primary IAV infection. The rise of antibiotic resistance in bacterial pathogens, such as MRSA, is a further threat with respect to enhancement of IAV morbidity and mortality. In conclusion, we report the first example of a secreted staphylococcal factor that enhances IAV replication and that could represent a target for combination therapy to reduce the severity of IAV-S. aureus coinfection. In addition, the novel proviral activity could be applied to address global IAV vaccine shortages which are a major public health concern in the light of the threat of a global pandemic.

## MATERIALS AND METHODS

### Tissue culture.

Madin-Darby canine kidney (MDCK) cells were maintained in Dulbecco’s modified Eagle’s medium (DMEM) (Millipore-Sigma, United Kingdom) with 5% (vol/vol) fetal calf serum (FCS) (Invitrogen, United Kingdom) and 1% (vol/vol) penicillin/streptomycin/glutamine (PSG) (Invitrogen, United Kingdom) at 37°C and 5% CO_2_. A549 and DF1 cells were maintained in DMEM–10% (vol/vol) FCS–1% (vol/vol) penicillin/streptomycin (PS) (Invitrogen, United Kingdom). Chicken embryo fibroblast (CEF) cells were isolated as previously described (57) with some modifications. Briefly, macerated 10-day-old embryos were incubated in trypsin/EDTA for 30 min at 37°C and 5% CO_2_ and passed through a 100-μm-pore-size cell strainer (GE Healthcare, United Kingdom) to yield a single-cell suspension. Freshly isolated cells were maintained in M199 medium (Millipore-Sigma, United Kingdom)–4% (vol/vol) FCS–1% PS. CEF cells were used up to passage 6. Primary normal human lung fibroblast (NHLF) cells were purchased from Lonza, United Kingdom, and maintained in fibroblast growth medium, as recommended by the manufacturer. HBTECs were purchased from ATCC and cultured in airway epithelial cell basal medium supplemented with a bronchial epithelial cell growth kit (ATCC, USA). Cells were used at 80% confluence for infections.

### Influenza A virus.

PR8, A/Udorn/307/72, A/Mallard/Netherlands/10/99, the PR8 MUd 7:1 reassortant between PR8 and A/Udorn/307/72, and a 6:2 reassortant between PR8 and A/California/07/2009 strain IAVs were generated from plasmid clones by reverse genetics (30, 58–60). Other strains of IAV were available in the laboratory collection (61) or generously supplied by Wendy Barclay (62). For generation of infectious IAV stocks, a multiplicity of infection (MOI) of 0.01 was used to infect MDCK cells for 1 h at 37°C and 5% CO_2_. Cells were washed, serum-free medium–2.5 μg/ml N-acetyl trypsin (NAT) (Millipore-Sigma, United Kingdom) was added, and infections were allowed to proceed for 48 h. Supernatant was harvested, centrifuged at 4,000 × *g* for 10 min, and stored at −80°C until further use. Egg-grown stocks were generated by infection of 11-day-old embryonated hen’s eggs (Henry Stewart, United Kingdom) with 100 PFU of virus. At 2 days postinfection, eggs were chilled and allantoic fluid was harvested, centrifuged twice at 4,000 × *g* for 10 min, and stored at −80°C until further use. Infectious viral titers were determined by plaque assay on MDCK cells, under an agarose overlay (63). To obtain a PR8 stock with uncleaved HA, infection was performed at an MOI of 3. After the inoculum was removed, cells were washed twice with phosphate-buffered saline (PBS), subjected to a 1-min wash using acid (10 mM HCl, 150 mM NaCl [pH 3]), and washed 3 times with PBS, after which serum-free medium without trypsin was added. Supernatant was harvested at 24 hpi and virus partially purified by ultracentrifugation as described below.

### Bacterial growth.

The bacterial strains used in this study are listed in Table S2 in the supplemental material. S. aureus isolates were grown overnight (O/N) on tryptic soya agar (TSA) or in tryptic soya broth (TSB) at 37°C with shaking at 200 rpm unless otherwise stated. E. coli isolates were grown on Luria-Bertani (LB) agar or in LB broth as described above. Where appropriate, medium was supplemented with antibiotics—ampicillin at 100 μg/ml, erythromycin at 10 μg/ml, or chloramphenicol at 12 μg/ml.

10.1128/mBio.00975-20.8 TABLE S2 Bacterial strains used in this study. Download Table S2, DOCX file, 0.03 MB. Copyright © 2020 Goncheva et al. 2020 Goncheva et al. This content is distributed under the terms of the Creative Commons Attribution 4.0 International license.

### Strain construction.

For complementation of Tn insertion mutants, full-length genes were amplified with primer pair Lip1 F and Lip 1 R and primer pair Lip 2 F and Lip 2 R (Table S3) and ligated into the pALC2073 vector (64). Plasmids were isolated with a Qiagen Spin Miniprep kit (Qiagen, United Kingdom) and transformed into S. aureus strain RN4220, from which they were transferred to appropriate S. aureus recipient strains by generalized transduction performed with phage 80α (65).

10.1128/mBio.00975-20.9 TABLE S3 Primer sequences used in this study. Download Table S3, DOCX file, 0.02 MB. Copyright © 2020 Goncheva et al. 2020 Goncheva et al. This content is distributed under the terms of the Creative Commons Attribution 4.0 International license.

For the generation of recombinant proteins, the lipase 1 and lipase 2 genes, without their signal peptide sequences, were amplified as described above, using primer pair rLip1 F and rLip1 R and primer pair rLip2F and rLip2R (Table S3), respectively, and inserted into pET15b vector, prior to transformation into E. coli DH5α. Plasmid was isolated and freshly transformed into E. coli BL21(DE3) cells prior to each induction. For site-directed mutagenesis, plasmid pET15b::lipase 1 was used with a QuikChange Lightning kit (Agilent Technologies, United Kingdom) per the manufacturer’s instructions.

### Protein isolation and expression.

For SEC, overnight (O/N) cultures in TSB were centrifuged at 4,000 × *g* for 15 min and filtered through a 0.45-μm-pore-size filter (Millipore, United Kingdom) and the supernatant was concentrated (5-to-7-fold) by the use of Amicon Ultra centrifugal units (10-kDa cutoff) to reach a total volume of 10 ml. Volumes (10 ml) of the concentrated supernatant were then loaded on a Superdex 75-pg size exclusion column (GE Healthcare, United Kingdom) equilibrated with 50 mM Tris (pH 7.5). Fractions (10 ml) were collected at a flow rate of 2.5 ml/min. Following chromatography, protein-containing fractions were subjected to ethanol precipitation. Briefly, 4 volumes of 100% ethanol were added to each fraction, and the fraction was frozen at −20°C for 4 h, centrifuged at 4,000 × *g* for 45 min, and resuspended in 1/10 the original volume in 50 mM Tris (pH 7.5). For SEC of complemented strains, cultures were grown in TSB to an optical density at 600 nm (OD_600_) of 0.6 to 0.8, induced with 125 ng/ml of tetracycline O/N, and processed as described above.

IEC was performed on S. aureus USA300 WT SEC fractions 2 to 4, which were combined and separated on a SP Sepharose column (GE Healthcare, United Kingdom) equilibrated with 50 mM Tris (pH 8.0). An elution gradient of 0% to 50% buffer (20 mM Tris [pH 8.0]–1 M NaCl) was used at a flow rate of 2.5 ml/min, and 5-ml fractions were collected. Fractions were subjected to ethanol precipitation as described above prior to use.

Recombinant protein was purified from cultures of E. coli BL21. Briefly, 1-liter cultures were grown in LB with 100 μg/ml ampicillin until an OD_600_ of 0.6 to 0.8 was reached. Cultures were then induced with 1 mM IPTG (isopropyl-β-d-thiogalactopyranoside) for 4 h, pelleted, and frozen at −20°C. When required, pellets were defrosted, resuspended in 50 ml lysis buffer (50 mM NaH_2_PO_4_, 300 mM NaCl, 10 mM imidazole) with complete protease inhibitor (Roche, United Kingdom), passed through a One-Shot cell disruptor (Constant Systems, Northants, United Kingdom) at 30,000 lb/in^2^, centrifuged at 4,000 × *g* for 30 min, and passed through a 0.45-μm-pore-size filter. Proteins were purified by immobilized metal affinity chromatography (IMAC) performed with a FF Crude nickel-nitrilotriacetic acid (Ni-NTA) column (GE Healthcare, United Kingdom). The flow rate was 2.5 ml/min, with an elution gradient of 0% to 100% buffer (50 mM NaH_2_PO_4_, 300 mM NaCl, 250 mM imidazole) over 30 min. Protein was dialyzed in 50 mM Tris (pH 7.5) using Spectra/Por Float-a-Lyzer tubing with an 8,000-to-10,000-molecular-weight cutoff (Spectrum Laboratories, CA, USA). Relative protein concentrations were estimated by the use of a bicinchoninic acid (BCA) protein assay kit (Novagen, United Kingdom) following the manufacturer’s instructions.

For studies performed with IEC or SEC fractions, CEF cells were infected at MOI 0.01 and 50-μl volumes of ethanol-concentrated fractions were added to 1 ml of serum-free media. For studies performed with recombinant protein, 50-μl volumes of concentrated stock were added to 1 ml of serum-free medium to reach the final indicated concentration. Infections performed at MOI 0.01 were harvested at 48 h and those at MOI 3 at 24 h.

### Egg infections.

Embryonated hen’s eggs (Henry-Stewart, United Kingdom) (10 days old) were used for all infections performed with rlipase 1. The eggs were infected with 100 PFU and 100 nM rlipase 1 (assuming an allantoic fluid volume of 10 ml) or with buffer to reach a total volume of 100 μl. The eggs were incubated for a further 48 h at 35°C and chilled O/N at 4°C, and allantoic fluid ws harvested. HA assays were performed as previously described (30). For partial purification of virus, the allantoic fluid from 4 or 5 eggs was pooled, clarified twice by centrifugation at 2,000 × *g* for 5 min, and loaded onto a 30% sucrose cushion. Centrifugation was carried out at 4°C and 28,000 rpm for 3 h on a Beckman XL-71 machine (Beckman, United Kingdom) (SW28 rotor). Supernatant and sucrose were aspirated, and the tube was filled with PBS, followed by centrifugation at 28,000 rpm at 4°C for 1 h. PBS was removed, 300 μl of PBS ws added, and the pellet was allowed to lift at 4°C overnight. All samples were then equalized to the same volume before treatment was performed with N-glycosidase F (PNGase F; New England Biolabs), according to the manufacturer’s protocol.

### RNA isolation and quantitative real-time PCR (qRT-PCR).

CEF cells were infected with PR8 at an MOI of 3 or were subjected to mock infection. Addition of rlipase 1 or rlipase 1 S408A was performed to reach a final concentration of 300 nM after inoculum removal, per the standard protocol. At 6 h and 8 h postinfection, the supernatant was harvested and the cells were washed twice with PBS and lysed in RLT buffer with 143 μM β-mercaptoethanol (Qiagen, United Kingdom) (500 μl/well for a 6-well plate). Samples were processed with a QIAshredder (Qiagen, United Kingdom), and RNA was extracted with an RNeasy kit (Qiagen, United Kingdom), according to the manufacturer’s instructions, with a DNase step included on the column. cDNA was generated with a SuperScript VILO cDNA synthesis kit (Thermo Fisher, United Kingdom) in a 100-μl volume, per the manufacturer’s instructions, using 500 ng RNA per cDNA reaction. A 5-μl volume of a 1-in-4 dilution of the cDNA was used for quantitative PCRs (qPCRs) with FastStart universal SYBR green master mix (Roche, United Kingdom). qPCRs were performed in 20-μl reaction volumes with 400 nM forward primer and 500 nM reverse primer for the M1 and chicken actin genes (Table S2) and 300 nM forward primer and 300 nM reverse primer for the PB1 gene (Table S3). qPCR conditions consisted of 1 cycle at 95°C for 10 min and 40 cycles of 95°C for 10 s followed by 50 s at annealing temperature (Table S4), with fluorescence acquisition performed at the annealing step, on a Rotor-Gene Q PCR machine (Qiagen, United Kingdom). Analysis was performed in triplicate, with the average value taken and normalized to chicken actin gene levels to give threshold cycle (Δ*C_T_*) values.

### Western blotting analyses.

CEF cells in 6-well plates were infected with PR8 at MOI of 1 and treated with 300 nM rlipase 1 as the standard. At 6, 8, and 10 h postinfection (hpi), cells were washed with PBS and lysed in 200 μl 2× Laemmli buffer (Sigma-Aldrich, United Kingdom). Protein was separated on a 12% SDS-PAGE gel and transferred to a nitrocellulose membrane (GE Healthcare, United Kingdom) by the use of a Trans-Blot Turbo blotting system (Bio-Rad, United Kingdom), according to the manufacturer’s instructions. Membranes were incubated for 60 min in PBS–0.1% Tween 20 (Sigma-Aldrich, United Kingdom) (PBST)–5% (wt/vol) dried milk (Sigma-Aldrich, United Kingdom) and washed 3 times with PBST. Primary antibody mixed in PBST was added, followed by incubation for 2 h at room temperature or O/N at 4°C. For detection of viral proteins, in-house rabbit sera (PB1, PB2, M1, and whole anti-PR8 virus sera for HA_0_), 1:500 anti-NP, 1:250 mouse monoclonal antibody 14C2 (M2), and 1:1,000 goat polyclonal anti-IAV H1N1 virus antibody (AbD Serotec 5315-0064) were used as primary antibodies. Tubulin was detected with a rat anti-tubulin antibody (Bio-Rad, United Kingdom) (1:1,000). HA from the H1N1 2009 pandemic virus (pH1N1) was detected with a rabbit polyclonal anti-swine H1 HA antibody (Ab91641; Abcam) (1:500). Rlipase 1 was used for the generation of a rabbit polyclonal antibody (Eurogentec, Belgium), using a proprietary 28-day program. The antibody was used at a 1:3,300 dilution to detect lipase 1 expression. Membranes were then washed 3 times for 5 min each time in PBST followed by incubation for 45 to 60 min with secondary antibody (donkey anti-rabbit antibody [IRDye 800RD] or goat anti-rat antibody [IRDye 680RD]; Li-Cor, United Kingdom) diluted in PBST before a further 5 or 6 washes with PBST and imaging on an infrared scanner (Li-Cor, United Kingdom) were performed.

### Confocal and scanning electron microscopy.

CEF cells were seeded at a density of 1 × 10^5^ cells/well on glass coverslips the day prior to infection. Cells were infected at an MOI of 3, and rlipase 1 was added to reach a concentration of 300 nM immediately after inoculum removal. For confocal microscopy, cells were fixed with 4% paraformaldehyde for 20 min at 8 hpi. Cells were washed 3 times with PBS–1% FBS and incubated with rabbit anti-PR8 antibody at 1:500 for 1 h at room temperature. Following 3 washes with PBS–1% FBS, cells were incubated with Alexa Fluor 488-conjugated anti-rabbit secondary antibody (Thermo Fisher [A-21206]) (1:1,000) and DAPI (4′,6-diamidino-2-phenylindole) (Invitrogen) (1:10.000) for 45 min at room temperature. Cells were washed as described above, and the glass coverslips were mounted on microscope slides using approximately 5 μl of ProLong antifade reagent (Invitrogen). The cells were imaged in a Leica LSM710 confocal microscope using a 63× lens objective. Images were collected as z-stacks across the depth of the cell membrane, generally in 0.45-μm increments, and are presented as maximum intensity projections. For counting the number of infected cells, a minimum of 60 cells were scored for the presence or absence of viral filaments. For measurement of filament length, a minimum of 60 filaments were measured using Image J (66). For scanning electron microscopy, cells were fixed with 3% glutaraldehyde–0.1 M sodium cacodylate buffer (pH 7.3) O/N and then washed 3 times for 10 min each time with 0.1 M sodium cacodylate buffer. Samples were then postfixed in 1% osmium tetroxide–0.1 M sodium cacodylate buffer for 45 min. A further 3 washes (10 min each) were performed in 0.1 M sodium cacodylate buffer. Cells were dehydrated in graded concentrations of acetone (once each at 50%, 70%, and 90% and 3 times at 100%) for 10 min each time followed by critical point drying using liquid carbon dioxide. After mounting of the specimens on aluminum stubs with carbon tabs attached, they were sputter coated with 20-nm-diameter gold palladium particles and viewed using a Hitachi S-4700 scanning electron microscope.

### Lipase assays.

Lipase assays were performed using purified recombinant protein as previously described (67). Individual reaction mixtures contained 36 μl of Tween 20 as the substrate (diluted 1 in 10 in 20 mM Tris-HCl), 30 μl of 100 mM CaCl_2_, 84 μl of 20 mM Tris (pH 8), and 50 μl recombinant protein at the indicated concentrations. The reaction mixtures were incubated at 37°C in an Optima plate reader (Fluostar, United Kingdom), with shaking performed every 3 min. Optical density measurements at OD_495_ were obtained every 5 min for a period of 24 h.

### Mouse infections.

All work involving animals was carried out under a United Kingdom Home Office license according to the Animals (Scientific Procedures) Act of 1986. Female BALB/c mice (10 to 12 weeks of age) were anaesthetized with isoflurane (Merial Animal Health Ltd.) and intranasally infected with virus (10 PFU) or bacteria (1 × 10^7^ CFU) in a mixture with 40 μl PBS (Gibco, United Kingdom). Mice were weighed daily and scored for visual signs of clinical disease, including inactivity, ruffled fur, and labored breathing. Clinical scores were quantitated on a scale of 0 to 3, and daily scores were added together. Animals that had exhibited severe clinical signs or had lost 25% to 30 % of their original body weight were euthanized by CO_2_ asphyxiation. Lungs were removed and homogenized in PBS in a Qiagen Tissue Lyser II instrument run at 28 shakes/s for 4 min (2 runs of 2 min). The resulting lysate was centrifuged at 3,000 × *g* for 5 min and supernatant collected. Viral titers were determined by a standard plaque assay performed on MDCK cells.

### Statistical methods.

Statistical analysis was performed with GraphPad Prism 7 or GraphPad Prism 8 software (GraphPad, USA).
