# Supplementary figures and images for: Staphylococcus aureus Lipase 1 Enhances Influenza A Virus Replication
Source: mBio. 2020 Jul 7;11(4):e00975-20. doi: 10.1128/mBio.00975-20 (PMC7343990; doi:10.1128/mBio.00975-20)

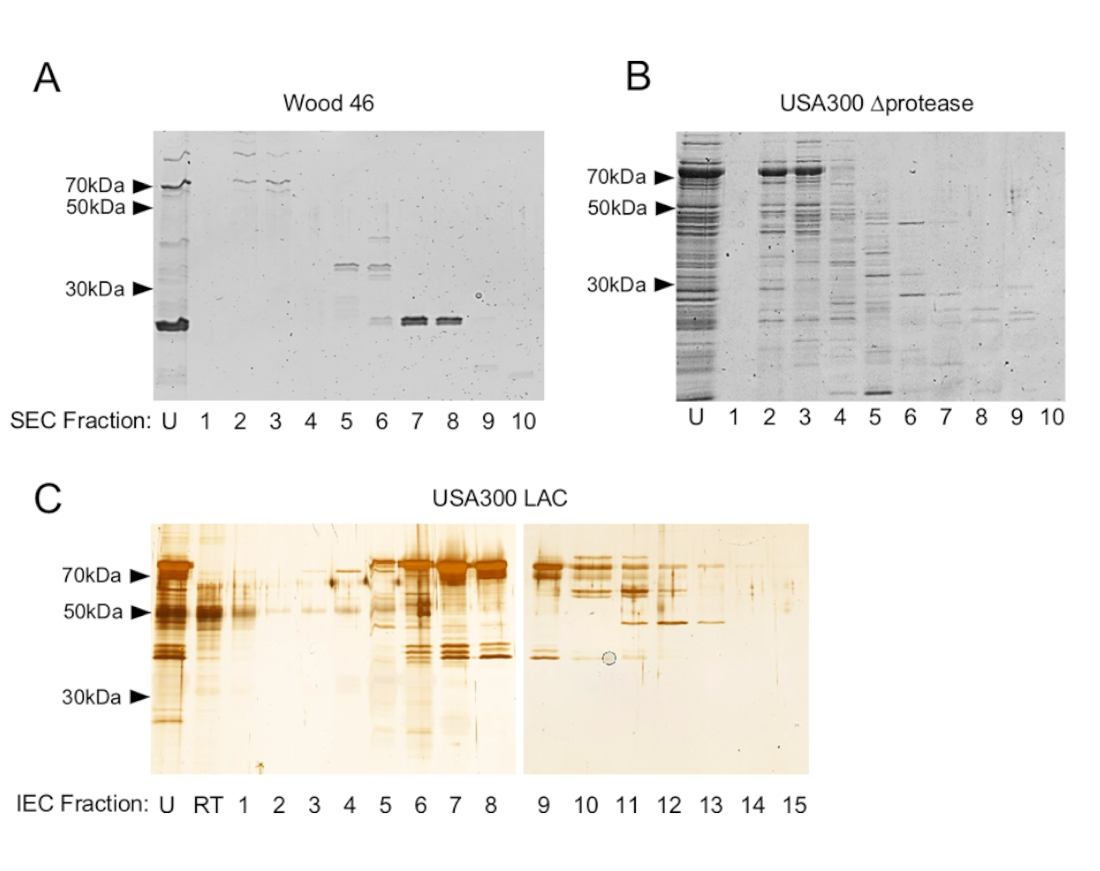

Supplement: FIG S1 [file mBio.00975-20-sf001.tif]

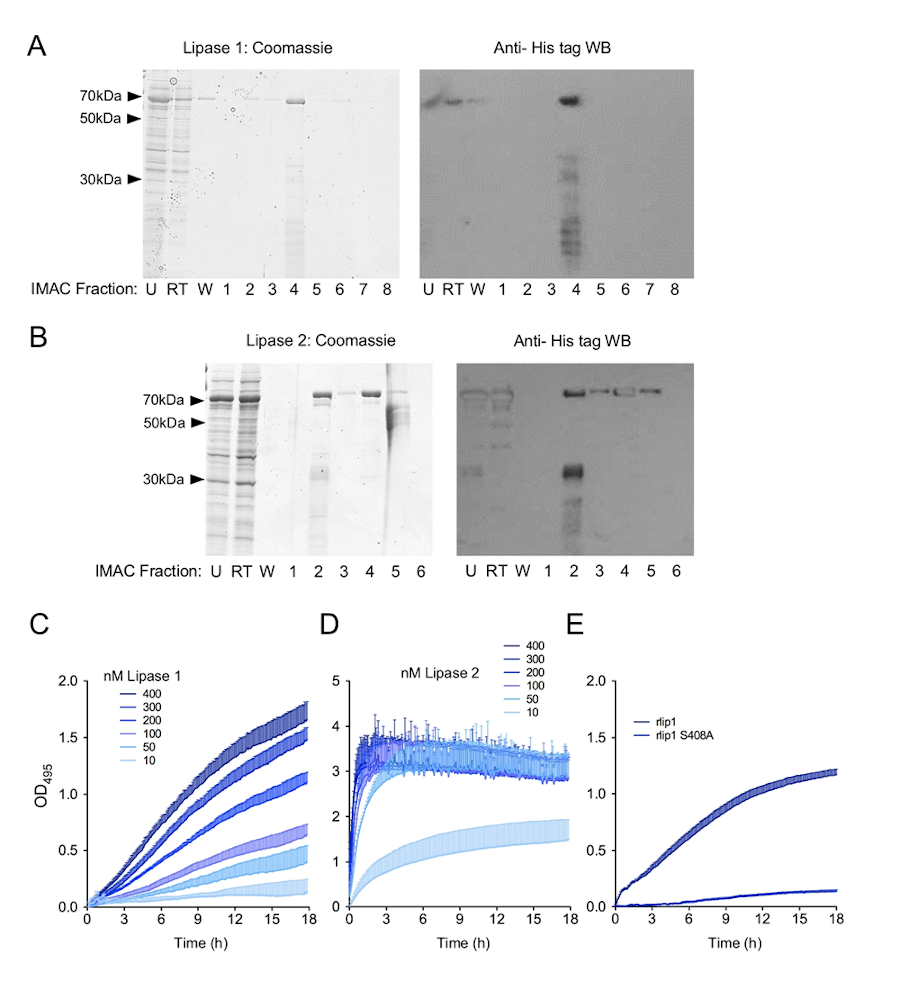

Supplement: FIG S2 [file mBio.00975-20-sf002.tif]

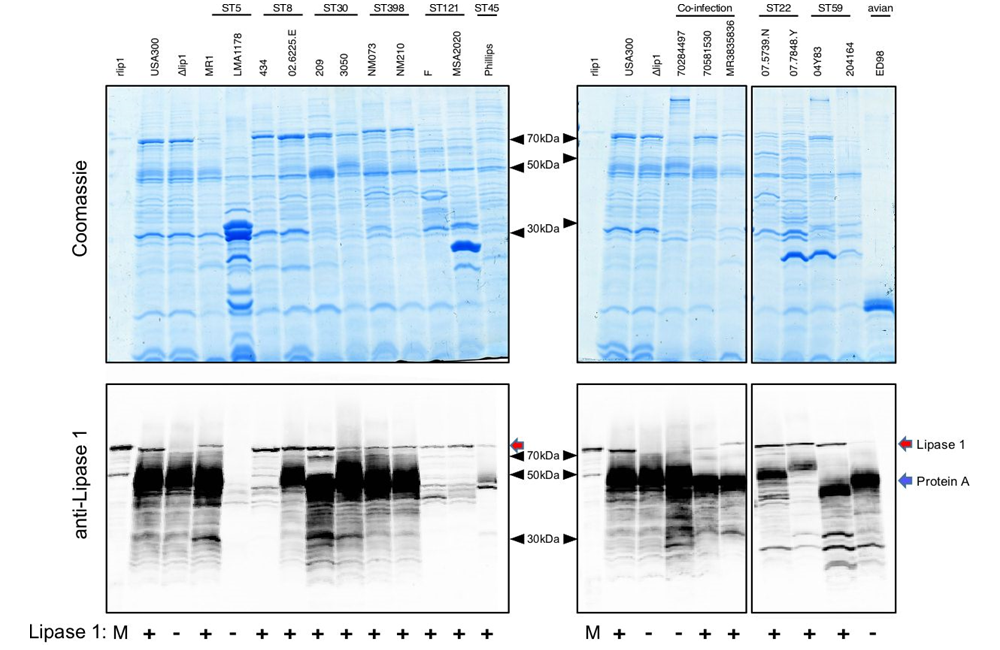

Supplement: FIG S3 [file mBio.00975-20-sf003.tif]

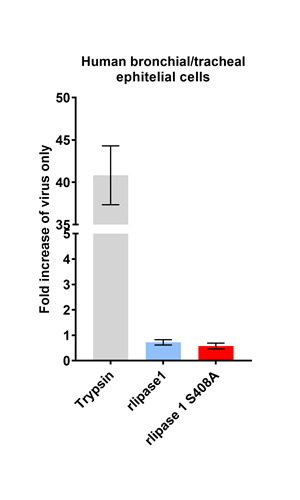

Supplement: FIG S4 [file mBio.00975-20-sf004.tif]

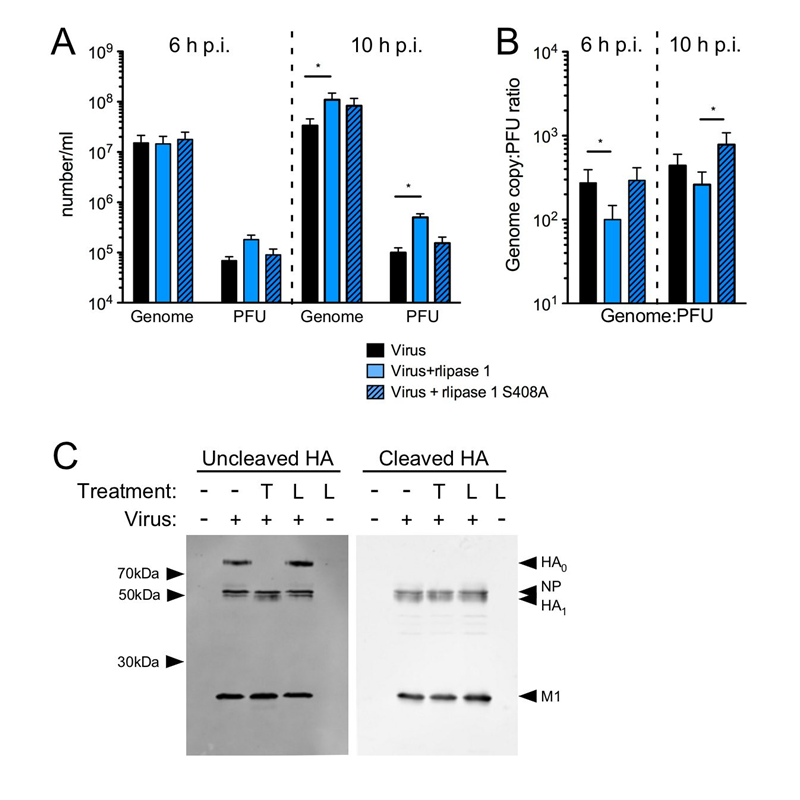

Supplement: FIG S5 [file mBio.00975-20-sf005.tif]

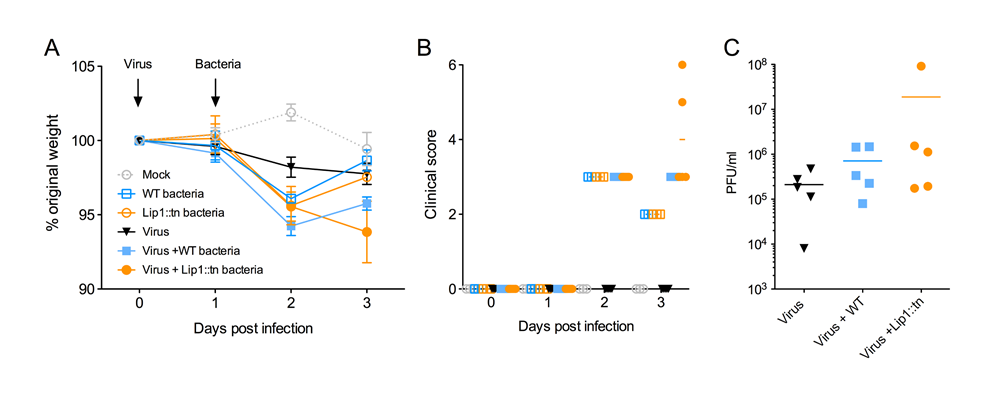

Supplement: FIG S6 [file mBio.00975-20-sf006.tif]
