# Supplementary material for: Staphylococcus aureus Lipase 1 Enhances Influenza A Virus Replication
Source: mBio. 2020 Jul 7;11(4):e00975-20. doi: 10.1128/mBio.00975-20 (PMC7343990; doi:10.1128/mBio.00975-20)
Supplement: TABLE S1 [file mBio.00975-20-st001.docx]

| Fraction number | Protein name (Accession number) | Mass (Da) | Score^a^ | Number of peptides^b^ |
| --- | --- | --- | --- | --- |
| 4 | Lipase 2 (SAUSA300_0320) | 76341 | 1651 | 20 |
|  | Lipase 1 (SAUSA300_2603) | 76629 | 1297 | 27 |
|  | N-acetylmuramoyl-L-alanine amidase domain-containing protein (SAUSA300_2256) | 69269 | 460 | 12 |
|  | Surface protein G (SATW20_RS14385) | 178419 | 96 | 4 |
|  | Immunoglobulin G-binding protein A (SAUSA300_0113) | 56403 | 86 | 3 |
| 5 | Lipase 1 (SAUSA300_2603) | 76629 | 8059 | 31 |
|  | Lipase 2 (SAUSA300_0320) | 76341 | 6558 | 25 |
|  | Glyceraldehyde-3-phosphate dehydrogenase (SAUSA300_1633) | 36372 | 360 | 3 |
|  | Enolase (SAOUHSC_00799) | 47145 | 301 | 2 |
|  | N-acetylmuramoyl-L-alanine amidase domain-containing protein (SAUSA300_2579) | 69269 | 301 | 2 |
| 6 | Lipase 2 (SAUSA300_0320) | 76341 | 2468 | 17 |
|  | Lipase 1 (SAUSA300_2603) | 76629 | 699 | 10 |
| 7 | Lipase 2 (SAUSA300_0320) | 76341 | 13650 | 28 |
|  | Lipase 1 (SAUSA300_2603) | 76629 | 1885 | 7 |

Table S1 – Identities of proteins present in pro-viral fractions. The proteins identified in four pro-viral fractions of *S. aureus* supernatant by LC MS/MS. Hits with an overall score > 40 and with more than 2 peptides are shown.
