# Supplementary material for: Staphylococcus aureus Lipase 1 Enhances Influenza A Virus Replication
Source: mBio. 2020 Jul 7;11(4):e00975-20. doi: 10.1128/mBio.00975-20 (PMC7343990; doi:10.1128/mBio.00975-20)
Supplement: TABLE S2 [file mBio.00975-20-st002.docx]

Table S2 Bacterial strains used in this study.

| Bacterial strain | Phenotype | Source |
| --- | --- | --- |
| AH1263 | Wild type USA300 LAC | (1) |
| AH1919 | No production V8, Aureolysin, Staphopain A, Staphopain B, splA, splD, splC, splD, splE, splF | (1) |
| *S. aureus* USA300 Lipase 1*::Bursa* | *Tn* insertion mutant of lipase 1 | (2) |
| *S. aureus* USA300 Lipase 2*::Bursa* | *Tn* insertion mutant of lipase 2 | (2) |
| *S. aureus* USA300 N-acetylmuramoyl-L-alanine amidase domain-containing protein *::Bursa* | *Tn* insertion mutant of N-acetylmuramoyl-L-alanine amidase domain-containing protein | (2) |
| *S. aureus* USA300 Glyceraldehyde-3-phosphate dehydrogenase*::Bursa* | *Tn* insertion mutant of Glyceraldehyde-3-phosphate dehydrogenase | (2) |
| *S. aureus* USA300 Lipase 1*::Bursa* + pALC::lipase1 | *Tn* insertion mutant of lipase 1 complemented with a copy of lipase 1 on an inducible plasmid | This study |
| *S. aureus* USA300 Lipase 2*::Bursa* + pALC::lipase2 | *Tn* insertion mutant of lipase 2 complemented with a copy of lipase 2 on an inducible plasmid | This study |
| Wood 46 | Wild type | (3) |
| *S. aureus* RN4220 with pALC::lipase1 | RN4220 containing an inducible plasmid encoding lipase 1 | This study |
| *S. aureus* RN4220 with pALC::lipase2 | RN4220 containing an inducible plasmid encoding lipase 2 | This study |
| MR1 | ST5 wound isolate from Poland | (4) |
| LMA1178 | ST5 invasive infection isolate from France | (5) |
| 434 | ST8 blood isolate from the UK | (6) |
| 02.6225.E | ST8 perianal isolate from the UK | Scottish reference laboratory |
| 209 | ST30 infective endocarditis isolate from the UK | (6) |
| 3050 | ST30 commensal isolate | (6) |
| NM073 | ST398 isolate from the USA | Lab archive |
| NM210 | ST398 isolate from Spain | Lab archive |
| F | ST121 isolate from Spain | (7) |
| MSA2020 | ST121 scalded skin syndrome isolate from France | (8) |
| Phillips | ST45 isolate | Lab archive |
| 70284497 | IAV – *S. aureus* co-infection isolate from Germany | (9) |
| 70581530 | IAV – *S. aureus* co-infection isolate from Germany | (9) |
| MR3835836 | IAV – *S. aureus* co-infection isolate from the UK | Edinburgh Royal infirmary |
| 07.5739.N | ST22 spleen isolate from the UK | Scottish reference laboratory |
| 07.7848.Y | ST22 blood isolate from the UK | Scottish reference laboratory |
| 04Y83 | ST59 urine isolate from Taiwan | (10) |
| 204164 | ST59 biopsy isolate from Taiwan | (10) |
| ED98 | ST5 broiler chicken isolate from Ireland | (4) |
| E. coli DH5α | Cloning strain | Novagen, UK |
| *E. coli* DH5α+ pALC2073 | DH5α strain containing an empty inducible plasmid | This study |
| *E. coli* DH5α + pALC::lipase1 | DH5α strain containing an inducible plasmid encoding lipase 1 | This study |
| *E. coli* DH5α + pALC::lipase2 | DH5α strain containing an inducible plasmid encoding lipase 2 | This study |
| Strataclone SoloPack™ | Cloning strain | Invitrogen, UK |
| *E. coli* DH5α + pET15b | DH5α strain containing empty pET15b | Novagen, UK |
| *E. coli* DH5α + pET15b::lipase1 | DH5α strain containing pET15b carrying the lipase 1 gene | This study |
| *E. coli* DH5α + pET15b::lipase2 | DH5α strain containing pET15b carrying the lipase 2 gene | This study |
| pET15b::lipase1 S408A | DH5α cells with a plasmid carrying the lipase 1 gene with a S408A substitution | This study |
| *E. coli* BL21 (DE3) | Expression strain | Invitrogen, UK |
| *E. coli* BL21 + pET15b::lipase1 S408A | BL21 cells with a plasmid carrying the lipase 1 gene with a S408A substitution | This study |

**Supplementary references**

1. Wörmann ME, Reichmann NT, Malone CL, Horswill AR, Gründling A (2011) Proteolytic cleavage inactivates the *Staphylococcus aureus* lipoteichoic acid synthase. *J Bacteriol* 193(19):5279–5291.

2. Fey PD, *et al*. (2013) A Genetic resource for rapid and comprehensive phenotype screening of nonessential *Staphylococcus aureus* genes. *MBio* 4(1). doi:10.1128/mBio.00537-12.

3. Tashiro M, Ciborowski P, Klenk H-D, Pulverer G, Rott R (1987) Role of Staphylococcus protease in the development of influenza pneumonia. *Nature* 325:536.

4. Lowder B V, *et al*. (2009) Recent human-to-poultry host jump, adaptation, and pandemic spread of *Staphylococcus aureus.* *Proc Natl Acad Sci* 106(46):19545 LP-19550.

5. Ben Zakour NL, Guinane CM, Fitzgerald JR (2008) Pathogenomics of the staphylococci: insights into niche adaptation and the emergence of new virulent strains. *FEMS Microbiol Lett* 289(1):1–12.

6. Feil EJ, *et al*. (2003) How clonal is *Staphylococcus aureus*? *J Bacteriol* 185(11):3307–3316.

7. Viana D, *et al*. (2015) A single natural nucleotide mutation alters bacterial pathogen host tropism. *Nat Genet* 47:361.

8. Fitzgerald JR, et al. (2006) Fibronectin-binding proteins of *Staphylococcus aureus* mediate activation of human platelets via fibrinogen and fibronectin bridges to integrin GPIIb/IIIa and IgG binding to the FcγRIIa receptor. *Mol Microbiol* 59(1):212–230.

9. Löffler B, *et al*. (2013) Pathogenesis of *Staphylococcus aureus* necrotizing pneumonia: the role of PVL and an influenza coinfection. *Expert Rev Anti Infect Ther* 11(10):1041–1051.

10. Ward MJ, *et al.* (2016) Identification of source and sink populations for the emergence and global spread of the East-Asia clone of community-associated MRSA. *Genome Biol* 17(1). doi:10.1186/s13059-016-1022-0.
