# Supplementary material for: Staphylococcus aureus Lipase 1 Enhances Influenza A Virus Replication
Source: mBio. 2020 Jul 7;11(4):e00975-20. doi: 10.1128/mBio.00975-20 (PMC7343990; doi:10.1128/mBio.00975-20)
Supplement: TABLE S3 [file mBio.00975-20-st003.docx]

Table S3 Primer sequences used in this study.

| Primer name | Sequence |
| --- | --- |
| Lip1 F | AAAAGGTACCATAGAGGTGCTGACAATGAT |
| Lip1 R | AAAAGAATTCTTATGCTTGCTTAGTATCAG |
| Lip2 F | AAAAGGTACCTACTTTTGAGGTGATTATATG |
| Lip2 R | AAAAGAATTCTTAACTTGCTTTCAATTGTGT |
| rLip1 F | AAAACATATGAAGCAAGTGAATATGGGAAATTCACA |
| rLip1 R | AAAACTCGAGTTATGCTTGCTTAGTATCAGTC |
| rLip2 F | AAAACATATGTCGGAAAAAACATCAACTAATGCAGCGGCAC |
| rLip2 R | AAAACTCGAGTTAACTTGCTTTCAATTGTGTTCCTTTAC |
| Lip1SDM F | GGTACACCTAGTTGGACATGCTATGGGCGGTCAAACGATA |
| Lip1SDM R | TATCGTTTGACCGCCCATAGCATGTCCAACTAGGTGTACC |
| qPCR Chicken actin F | GAATCCCAAAGCCAATCG |
| qPCR Chicken actin R | CCCAGAGTCAAGCACAATCC |
| qPCR PR8 M1 F | ctctctatcgtcccgtcagg |
| qPCR PR8 M1 R | gagcgtgaacacaaatccta |
| qPCR PR8 PB1 F | ggaacaggatacaccatgga |
| qPCR PR8 PB1 R | agtggyccatcaatcgggtt |
